# Crosslinking-Induced Corneal Endothelium Dysfunction and Its Protection by Topical Ripasudil Treatment

**DOI:** 10.1155/2022/5179247

**Published:** 2022-01-13

**Authors:** Xuemei Wang, Yanlin Zhong, Minghui Liang, Zhirong Lin, Huping Wu, Cheng Li

**Affiliations:** ^1^Eye Institute and Affiliated Xiamen Eye Center of Xiamen University, Xiamen, Fujian, China; ^2^Fujian Provincial Key Laboratory of Ophthalmology and Visual Science & Ocular Surface and Corneal Diseases, Xiamen University, Xiamen, Fujian, China

## Abstract

**Purpose:**

To investigate the changes of corneal endothelium under different crosslinking conditions and the protective effect of ripasudil.

**Methods:**

Corneal crosslinking groups were infiltrated with riboflavin and subsequently irradiated with 0.54 J/cm^2^ or 1.08 J/cm^2^ UVA, while noncrosslinking groups included neither UVA nor riboflavin treatment, only 1.08 J/cm^2^ UVA and only riboflavin treatment. Corneal opacity, variations in corneal endothelial cells, and corneal thickness of all groups were observed by slit lamp, *in vivo* confocal microscopy, and optical coherence tomography. Immunofluorescence staining and scanning electron microscopy were performed to evaluate changes in the structure and function of the corneal endothelium. The mice that received a corneal crosslinking dose of 1.08 J/cm^2^ were instilled with ripasudil to explore its protective effect on the corneal endothelium.

**Results:**

Treatment with UVA and riboflavin caused an increase in corneal opacity and corneal thickness and decreased endothelial cell density. Furthermore, treatment with UVA and riboflavin caused endothelial cell DNA damage and destroyed the tight junction and pump function of the endothelium, while riboflavin or the same dose of UVA alone did not affect the endothelium. Ripasudil reduced DNA damage in endothelial cells, increased the density of cells, and protected the endothelium's integrity and function.

**Conclusion:**

Riboflavin combined with UVA can damage the corneal endothelium's normal functioning. The corneal endothelium's wound healing is dose-dependent, and the ROCK inhibitor ripasudil maintains the endothelium's pump and barrier functions.

## 1. Introduction

Corneal crosslinking (CXL) therapy has been introduced as a minimally invasive treatment to prevent the development of keratectasia, which has completely changed the treatment of keratoconus and other corneal ectasias such as pellucid marginal degeneration and iatrogenic ectasia. CXL has also been used in the treatment of other diseases, such as bullous keratopathy, infectious ulcers, ulcerative keratitis, and other causes of corneal edema [1].

The fundamentals of CXL and the molecular processes of photooxidative CXL are combined with riboflavin as a photosensitizer in this treatment. The intention of collagen CXL with riboflavin/UVA light is to artificially enhance crosslinking in the corneal stroma to restore mechanical stability [2]. The procedure damages cell membranes, causing keratocyte death, in addition to the positive tissue stiffening impact [3, 4]. Riboflavin is nontoxic and can be used as a biological drug or a coloring agent in food processing [2]. Riboflavin serves both as a photosensitizer to promote corneal stiffening (crosslinking) by UVA and as a shield to reduce the level of UVA to below the cytotoxic threshold [5].

Regarding the adverse reactions caused by CXL, some studies have reported damage or inflammation of the corneal endothelium following CXL [6]. According to the previous studies [7], the endothelial phototoxic level is 0.35 mW/cm^2^, and a minimal corneal thickness of 400 *μ*m is required to ensure safe riboflavin/UVA CXL using the typical 3 mW/cm^2^ surface irradiance (5.4 J/cm^2^ surface dose) [8]. Although some preventive measures have been taken, postoperative corneal edema, suggestive of endothelial damage, has also been reported in thicker corneas [9]. Furthermore, despite some studies linking CXL to endothelial cell death, scientists have yet to definitively ascertain whether CXL alters the structure and function of the corneal endothelium in *vivo* models.

Since the use of a mouse model can increase knowledge regarding the basic cellular and molecular pathways activated by CXL therapy [10], studying different CXL protocols in mice is useful for understanding the physiological responses of different intensities of CXL and to determine the appropriate range of UVA fluence of CXL in mice. Previous studies have demonstrated that even when the UVA fluence is dropped to 0.09 J/cm^2^, a considerable corneal stiffening impact is still generated, and a fluence between 1.62 and 2.7 J/cm^2^ generates the least negative effects, such as scar formation and neovascularization [10, 11]. A CXL protocol for mice has previously been established [12]. Since the corneal thickness of mice differs from that of human, the treatment parameters were altered according to the Lambert-Beer law to make the relative UVA absorption of the mouse cornea equal to that of the human cornea in the Dresden protocol [13]. According to this calculation [14], when the radiation dosage to the mouse cornea is 1.53 J/cm^2^, the corresponding radiation dose to the human cornea is 5.4 J/cm^2^. However, white central scars in the cornea can even be found at a dose of 1.53 J/cm^2^ [10]. Since a dose of 1.53 J/cm^2^ may be a relatively high dose for mouse corneal crosslinking, we chose threshold UVA dosages below 1.53 J/cm^2^ to investigate potential damage to the corneal endothelium by combined riboflavin/UVA therapy.

Previous pioneering studies have shown that inhibiting ROCK signaling promotes corneal endothelial cell adhesion, migration, proliferation, and wound healing [15–20]. In human keratinocytes, RhoA/ROCK signaling is one of the regulators involved in oxidative damage and apoptosis, and blockade of RhoA/ROCK with a ROCK inhibitor can reduce the levels of DNA damage [21]. After destroying the central endothelium via transcorneal freezing, individuals with Fuchs endothelial corneal dystrophy (FECD) were given eye drops containing a ROCK inhibitor, which reduced corneal edema and increased visual acuity [22, 23]. In individuals with bullous keratopathy, cell-based treatments also assisted the reformation of the corneal endothelium layer [24]. Ripasudil hydrochloride hydrate (K-115) is a ROCK inhibitor that selectively inhibits ROCK1 and ROCK2, and a dose of 0.4 percent ripasudil has been licensed in Japan for the treatment of glaucoma [25]. Ripasudil has been recommended as an effective medicine for adjuvant therapy in FECD patients [26, 27], as well as a prospective therapeutic agent for retinal hypoxia neovascular diseases [28].

In the present study, we exploited the transparent property of the cornea and created a model that provided direct visualization of the cellular behavior in response to CXL *in vivo*, as determined by *in vivo* confocal microscopy and optical coherence tomography (OCT), monitoring the swelling of the cornea as a result of endothelial cell function and morphology. Moreover, we found that riboflavin plays an important role in mediating corneal endothelial damage. Since ROCK inhibitors have been reported to suppress oxidative damage, we tested the effect of ripasudil on corneal endothelium changes in CXL-induced mice.

## 2. Materials and Methods

### 2.1. Animals

58 SPF male C57/BL6 mice (6−8 weeks old) (Shanghai SLAC Experimental Animal Center, China) were utilized in this work. They were housed in a clean environment with a temperature of 24 ± 1°C, a relative humidity of 59 ± 9%, and a 12 h/12 h light/dark cycle. The Xiamen University Experimental Animal Ethics Committee authorized the research procedure, which complied with the ARVO Declaration on the Use of Animals in Ophthalmology and Visual Studies. The mice (*n* = 58) were randomly separated into two sets. For the first part of the study, 40 mice were divided into five groups: 1.08 J/cm^2^ UVA only, riboflavin only, 0.54 J/cm^2^ UVA plus riboflavin, 1.08 J/cm^2^ UVA plus riboflavin, and neither UVA nor riboflavin, to investigate their effect of independent variables on endothelium layer integrity. In a second assessment using 6 mice from each group, for the control and Ripa group, mice received 1.08 J/cm^2^ UVA plus riboflavin to examine ripasudil's pharmacological effectiveness in the mouse CXL model.

### 2.2. Corneal Crosslinking Procedure

Pentobarbital (40 mg/kg) was injected intraperitoneally for anesthesia, and proparacaine eye drops were given topically. Mechanical removal of corneal epithelium in the range of diameter 2 mm and 0.27% riboflavin solution diluted in sodium chloride (Avedro, USA) was applied to the deepithelialized corneas for 20 minutes. Subsequently, a UVA lamp would be used to irradiate the cornea at 365 nm with a fluence of 0.54 J/cm^2^ (9 mW/cm^2^ for 1 minute) or 1.08 J/cm^2^ (9 mW/cm^2^ for 2 minutes) (UVX 2000 system, IROC Innocross AG Co. Ltd., Switzerland). Following CXL, the corneal epithelial incision was treated with levofloxacin eye drops (Tobradex; Alcon Laboratories, Inc) three times a day until it healed.

### 2.3. Eye Drop Treatment

One drop of 0.4% ripasudil (Kowa Company, Tokyo, Japan) was topically instilled (2.5 *μ*L) four times daily in both eyes of six mice; PBS was instilled in both eyes of six further mice as a control.

### 2.4. Assessment of the Ocular Surface

Having followed CXL, all the corneas were checked daily using a slit light (BQ900H Haag-Streit, Bern, Switzerland). Corneal opacity scores were made depending on the sum of the scores of the several stated measures employed for this aim at postoperative 36 h, 4 d, 7 d, and 14 d. Corneal opacity scores were calculated according to the degree of edema in the central and peripheral part of the cornea.

### 2.5. *In Vivo* Confocal Microscopy

The mice were used in our trial following the administration of pentobarbital (40 mg/kg). The central corneal structure was examined by confocal laser scanning microscopy using a Heidelberg Retina Scanner III/Rostock Corneal Module (Heidelberg Engineer GmbH, Heidelberg, Germany). One drop of carbomer gel (Alcon Laboratories, Fort Worth, TX) was utilized prior to observation. By adjusting the controller, the cap center was extended to the center of the cornea, and the computer screen showed a digital image of the cornea. At least 10 images were photographed of each structure: the superficial epithelial layer, basal epithelial layer, stromal layer, and endothelial layer. All measurements were performed by a researcher who was blinded to the specific experimental conditions. The built-in software program was used to evaluate the density of endothelial cells.

### 2.6. Optical Coherence Tomography

Anterior segment images were taken using optical coherence tomography (OCT) (OPTOPROBE, England) at postoperative 36 h, 4 d, 7 d, and 14 d. The central corneal thickness was measured using inbuilt software.

### 2.7. Staining with Immunofluorescence

Frozen sections and corneal whole mounts were fixed with acetone at –20°C for 10 minutes and then incubated at 4°C overnight with primary antibodies for Texas Red–X phalloidin (1 : 150), anti-ZO-1 (1 : 150), anti-Na^+^/K^+^-ATPase (1 : 200), anti-*γ*-H2AX (1 : 400), and anti-8-OHdG (1 : 200). The next day, samples were incubated with Alexa Fluor 488-conjugated IgG (1 : 300) or Alexa Fluor 594-conjugated IgG (1 : 300) for 2 h at room temperature in the darkness. A laser confocal scanning microscope (Fluoview 1000, Olympus, Japan) had been used to investigate the immunofluorescence staining after three washes in PBS and counterstaining with DAPI (H-1200, Vector).

### 2.8. Ultrastructure of the Corneal Endothelium

The corneas of the 5 groups were fixed overnight in PBS (pH 7.4) containing 2.5% glutaraldehyde at 4°C, following which 4 mm × 2 mm pieces were produced without contact with the endothelium. Then, the ultrastructure of the corneal endothelium was observed by scanning electron microscope (SEM, JSM6390LV, JEOL, Tokyo, Japan) after dehydration, drying, and gold plating.

### 2.9. Statistical Analysis

Statistical analysis was performed by using the GraphPad Prism 8.0 program (GraphPad Software, Inc, San Diego, CA). Two-way ANOVA was used to compare symptoms at different time points. One-way ANOVA was used to compare multiple groups. The probability < 0.05 was considered statistically significant. All data is reported as mean ± SD.

## 3. Results

### 3.1. CXL Irradiation Causes Corneal Edema in Mice

**Figure 1:**
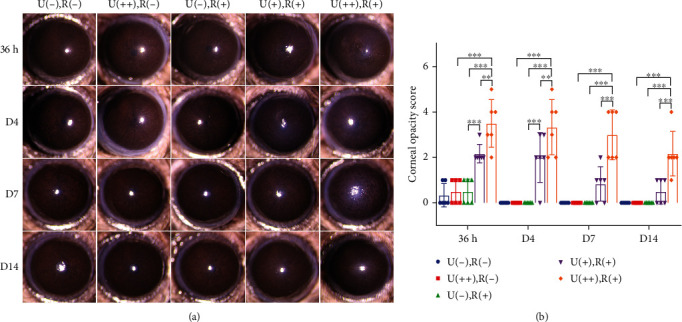
CXL irradiation causes corneal edema in mice. Representative slit-lamp images showing differences between the 5 different groups at time points corresponding to 36 h, 4 d, 7 d, and 14 d after CXL treatment. (a) Time-dependent changes in corneal opacity scores in the 5 groups. Data are expressed as the mean ± SD; *n* = 6. ^∗∗^*P* < 0.01; ^∗∗∗^*P* < 0.001. (b) U(-), R(-): neither UVA norriboflavin; U(++), R(+): 1.08 J/cm^2^ UVA; U(-), R(+): riboflavin only; U(+), R(+): 0.54 J/cm^2^ UVA plus riboflavin; U(++), R(+): 1.08 J/cm^2^ UVA plus riboflavin.

Slit-lamp examination revealed visible edema in the cornea of each CXL-treated group at posttreatment 36 h, while in the U(-), R(-), U(++), R(-), and U(-), R(+) groups, corneal edema was not obvious. In the U(+), R(+) group, corneal transparency was restored after 14 days, while the corneas remained edematous in the U(++), R(+) group (Figure 1(a)). In comparison with the U(++), R(-), or U(-), R(+) groups, the corneal opacity scores of CXL group were significantly higher at 36 h after treatment. In the U(+), R(+) group, scores reversed to the baseline on the 14th day, but in the U(++), R(+) group, the values remained high (Figure 1(b)).

### 3.2. CXL Irradiation Causes Alterations in Mouse Corneal Endothelial Cell Morphology

**Figure 2:**
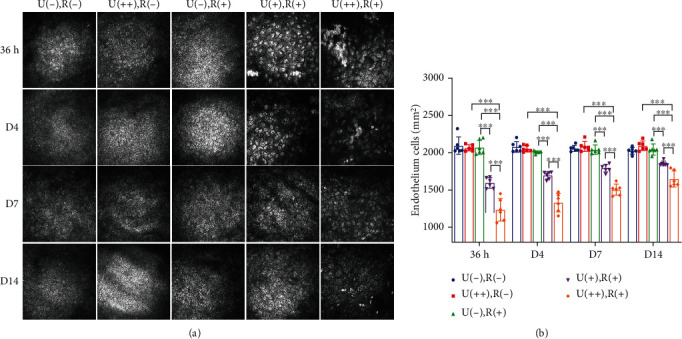
CXL irradiation causes morphological changes in mouse corneal endothelial cells and decreases cell density. (a) Representative confocal microscopy images showing differences in the endothelial cell layer between the 5 different groups at time points corresponding to 36 h, 4 d, 7 d, and 14 d after CXL treatment. (b) Cell density analysis of the 5 groups. Data are expressed as the mean ± SD; *n* = 6. ^∗∗∗^*P* < 0.001.

Mouse corneal endothelial cell morphology and density were assessed by *in vivo* confocal microscopy at various time points after CXL. The corneal endothelium showed a characteristic hexagonal monolayer with a regular size and shape in the U(-), R(-), U(++), R(-), and U(-), R(+) groups. However, CXL mice showed exacerbated morphological changes, such as increased cell size and loss of discernible cell borders as compared with the other three groups at the same recovery time points (Figure 2(a)).  Figure 2(b) depicts the variations in corneal endothelial cell density in each group over time. In the U(++), R(+) group, endothelial damage from 36 h to d14 persisted, and endothelial cell density was the lowest, being significantly less than that in the U(++), R(-), or U(-), R(+) groups.

### 3.3. CXL Irradiation Increases the Thickness of the Central Cornea by OCT

**Figure 3:**
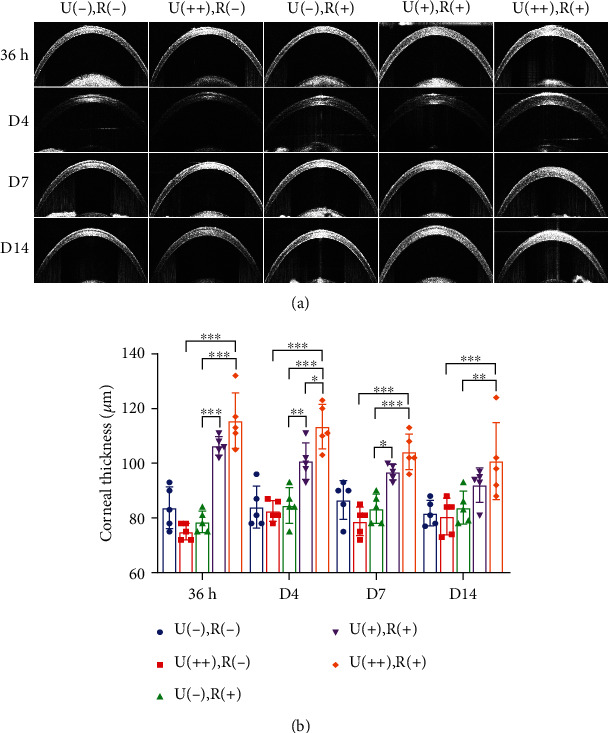
CXL irradiation modulates the thickness of the central cornea. (a) Representative OCT images of mouse corneas in the 5 different groups at time points corresponding to 36 h, 4 d, 7 d, and 14 d after CXL treatment. (b) Central corneal thickness analysis based on OCT images. Data are expressed as the mean ± SD; *n* = 5. ^∗^*P* < 0.05; ^∗∗^*P* < 0.01; ^∗∗∗^*P* < 0.001.

Corneal edema leads to an increase in central corneal thickness (CCT), which is a hallmark of endothelial cell functional impairment. In the U(++), R(-), and U(-), R(+) groups, the corneal thickness did not change significantly. OCT demonstrated that in the U(+), R(+), and U(++), R(+) groups, the corneal thickness was considerably higher than in the U(++), R(-), or U(-), R(+) groups at 36 h after CXL (Figures 3(a) and 3(b)). In the U(+), R(+) group, the thickness tended to be increased by 36 h, reaching significance but then returning to normal by day 14; however, the corneal thickness in the U(++), R(+) group did not recover even after 14 days (Figure 3(b)).

### 3.4. CXL Induces Na^+^/K^+^-ATPase Mislocalization and Disruption of Cytoskeletal Organization and Tight Junction Integrity

**Figure 4:**
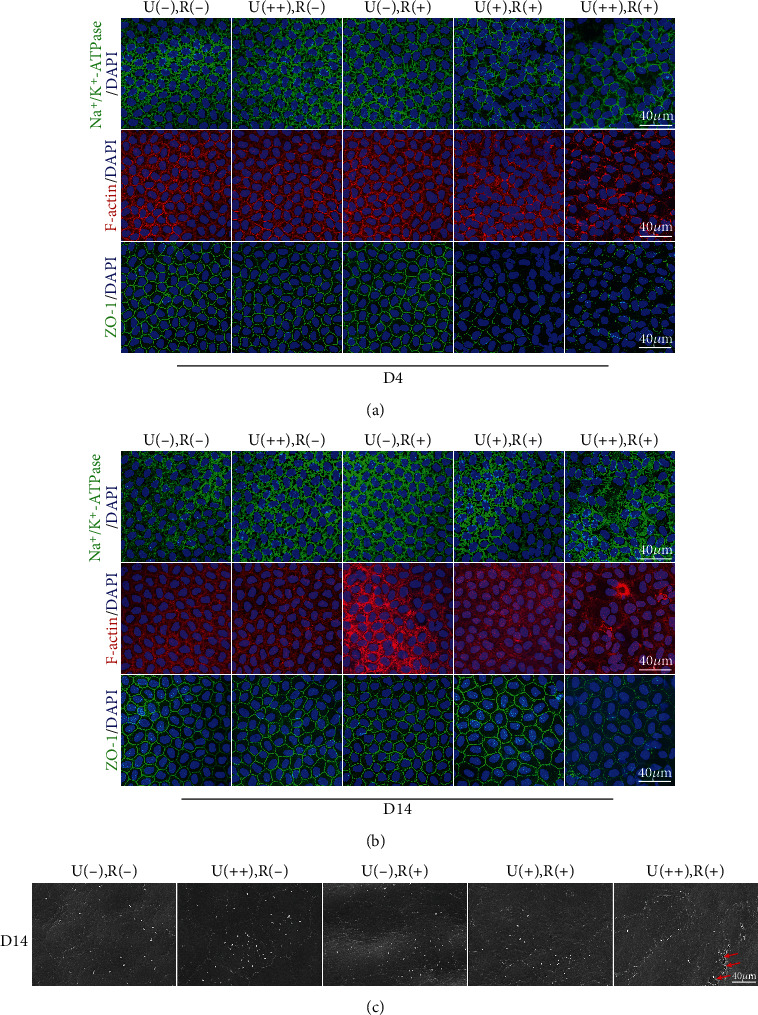
CXL irradiation destroys the integrity of tight junctions and disrupts the localization of Na^+^/K^+^-ATPase in corneal endothelial cells. (a, b) Representative confocal images of wholemount mouse central corneal endothelial cells detecting ZO-1, F-actin, and Na^+^/K^+^-ATPase localization on (a) day 4 and (b) day 14. On day 4, ZO-1, F-actin, and Na^+^/K^+^-ATPase staining of endothelial cells show a disrupted distribution of these markers in the U(+), R(+), and U(++), R(+) groups as compared with that in the other 3 groups. After 14 days, the normal distribution around the cell borders was restored in the U(+), R(+) group, but the recovery was still incomplete in the U(++), R(+) group. (c) Representative SEM images of the apical surface of central corneal endothelial cells on day 14. The microvilli on endothelial cells have almost disappeared, and the cell boundary between endothelial cells is discontinuous (arrows) in the U(++), R(+) group as compared with the U(++), R(-), or U(-), R(+) group.

Ion transporter proteins including as bicarbonate transporters, monocarboxylate transporters (MCT), and aquaporin water channels assist the pump function, which is primarily maintained by Na^+^/K^+^-ATPase [29]. In the U(++), R(-), and U(-), R(+) groups on day 4, Na^+^/K^+^-ATPase was uniformly and consistently expressed at the cell membrane, while its localized expression was disrupted and dispersed in the CXL groups (Figure 4(a)). The U(+), R(+) group's Na^+^/K^+^-ATPase localization began to restore to its usual distribution after 14 days, but the U(++), R(+) group's recovery was only partially complete (Figure 4(b)).

In the typical corneal endothelium, F-actin was found at the apical cell borders, resulting in a double-banded appearance. In the CXL groups, the double-banded structure had vanished by day 4 and the F-actin expression pattern was diffused (Figure 4(a)). The F-actin distribution in the U(+), R(+) group almost recovered after 14 days, but the F-actin distribution in the U(++), R(+) group was only partially restored and the cytoskeleton was partially rearranged (Figure 4(b)).

The corneal endothelium's barrier integrity is supported by cell-to-cell connections such as adhesion and tight junctions, which are primarily formed by ZO-1. ZO-1 produced a consistent hexagonal pattern in the non-CXL groups' corneal endothelium and was continually expressed along the cell boundary. On day 4, ZO-1 expression at the cell boundary in the CXL groups turned partial and discontinuous (Figure 4(a)); however, 14 days later, the distribution of ZO-1 in the U(+), R(+) group was substantially restored, while the distribution of ZO-1 in the U(++), R(+) group had not recovered (Figure 4(b)).

### 3.5. CXL Disrupts the Fine Structure of the Mouse Corneal Endothelial Cells

To obtain a more comprehensive overview of the CXL-induced changes in corneal endothelial cells, the fine structure of the posterior cornea was evaluated by SEM. In the U(-), R(-), U(++), R(-), and U(-), R(+) groups, the corneal endothelial cells appeared flat and hexagonal. In addition, sharply demarcated and interdigitating cellular borders, apical microvilli, and infrequent cilia were also observed. On day 14, the morphology of the corneal endothelial cells in the U(+), R(+) group had virtually recovered as compared to the U(-), R(+) group. The cell border between corneal endothelial cells became blurry in several locations in the U(++), R(+) group, indicating breakdown of cell-cell tight junctions (Figure 4(c), arrow).

### 3.6. CXL Induces DNA Damage in Mouse Corneal Endothelial Cells

**Figure 5:**
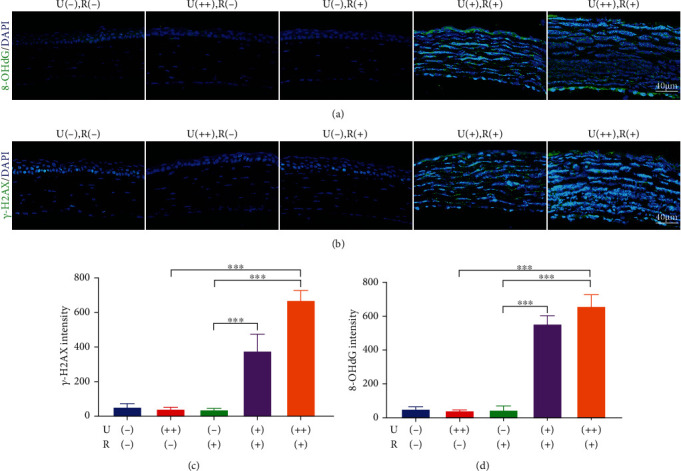
CXL irradiation causes DNA damage in corneal endothelial cells. (a) Representative confocal images of the mouse corneal endothelium with 8-OHdG staining at 36 h after CXL treatment. (b) Representative confocal images of the mouse corneal endothelium with *γ*-H2AX staining at 36 h after CXL treatment. (c) Mean immunofluorescence intensity of 8-OHdG in endothelial cells. (d) Mean immunofluorescence intensity of *γ*-H2AX in endothelial cells. Data are expressed as the mean ± SD; *n* = 3. ^∗∗∗^*P* < 0.001.

Intense immunostaining with anti-8-OHdG (8-hydroxy-2′-deoxyguanosine), a biomarker of DNA oxidation [30] occurring when DNA is oxidatively modified by ROS, was detected in the corneal endothelium in the CXL groups after 36 h. There was a significant increase in CXL-induced nuclear 8-OHdG in comparison with the U (++), R(-) or U(-), R(+) group, especially in the U(++), R(+) group (Figure 5(a)). H2AX is found in the nucleosome as a histone variant. H2AX is quickly phosphorylated at Ser139 within 1–3 minutes after DNA double-strand breaks, and the relative amount of phosphorylated H2AX molecules rises linearly with the severity of DNA damage [31, 32]. In the endothelial monolayer of the CXL groups, nuclei with very intense staining were evident, indicating the presence of DNA damage. The amount of *γ*-H2AX expression in the CXL groups was considerably greater than in the U(++), R(-) or U(-), R(+) groups. The punctate staining pattern clearly implies that corneal endothelial cells respond to nuclear DNA damage by producing DNA damage foci, and positive nuclear staining for H2AX phosphorylated at Ser139 gives evidence that corneal endothelial cells are capable of detecting nuclear DNA damage (Figure 5(b)).

### 3.7. Ripasudil Reduces Corneal Edema in CXL Mice and Protects the Corneal Endothelium from CXL-Induced Injury

**Figure 6:**
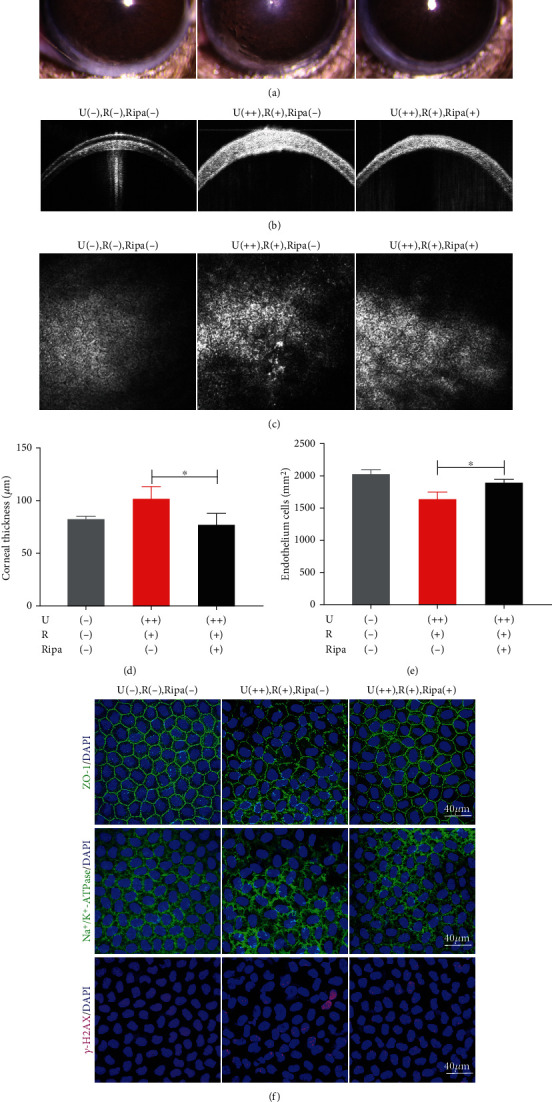
Ripasudil reduces corneal edema and exhibits protection of the corneal endothelium from CXL irradiation in mice. (a) Mouse corneal clarity was examined by slit-lamp microscopy with or without ripasudil treatment after 7 days of CXL treatment. (b) Representative OCT images of mouse corneas after 7 days of CXL treatment. (c) Representative *in vivo* confocal images of corneal endothelial cells after 7 days of CXL treatment. (d) Analysis of the corneal thickness based on the OCT image on day 7 after CXL irradiation. (e) Endothelial cell density analysis of corneas on day 7 after CXL irradiation. (f) Representative confocal images of wholemount mouse corneal endothelium with ZO-1, Na^+^/K^+^-ATPase, and *γ*-H2AX staining on day 7 after CXL irradiation. Data are expressed as the mean ± SD; *n* = 4. ^∗^*P* < 0.05. U(-), R(-), Ripa(-): no UVA, riboflavin, or ripasudil treatment; U(++), R(+), Ripa(-): 1.08 J/cm^2^ UVA plus riboflavin only; U(++), R(+), Ripa(+): 1.08 J/cm^2^ UVA, riboflavin, and ripasudil treatment.

It was observed that multiple daily administrations of ripasudil eye drops in mouse eyes for 7 days improved corneal opacification as compared with the U(++), R(+), Ripa(-) group (control) (Figure 6(a)). On day 7, the corneal thickness of the U(++), R(+), Ripa(+) mice was considerably lower than that of the control group (Figures 6(b) and 6(d)).

*In vivo* confocal microscopy revealed relatively minor cell body expansion and loose intercellular connections in the U(++), R(+), Ripa(+) group, in contrast to the loose and flaky shedding seen in the control group (Figure 6(c)). Ripa-treated corneal endothelial cells had a considerably higher density than those in the control group (Figure 6(e)). Wholemount immunofluorescence labeling of ZO-1, Na^+^/K^+^-ATPase, and -H2AX was done to further investigate the protective effect of ripasudil on corneal endothelial cells in CXL animals. The tight junction marker ZO-1 exhibited integrated structures with a defined boundary in the Ripa-treated group, but ZO-1 expression at the cell border was irregular, even absent, in the control group. Na^+^/K^+^-ATPase, a pump functional marker, exhibited regular and continuous expression around the cell border in the Ripa-treated group, which was similar to the non-CXL group. However, the expression of Na^+^/K^+^-ATPase became sparse and was absent in certain localized areas in the control group. *γ*-H2AX is an important marker of DNA damage. In the control group, some nuclei with intense staining were observed in the corneal endothelium on day 7; however, the Ripa-treated group showed no positive nuclei staining in the endothelium at the same time point (Figure 6(f)).

## 4. Discussion

CXL is a well-known therapy that is receiving increased popularity as a treatment choice for progressive keratoconus, especially considering its relatively low level of side effects. Despite the reported high safety profile, some reports indicate the possibility of corneal endothelial damage with obvious corneal edema after CXL treatment [33]. We used a mouse corneal crosslinking model to study endothelial cell changes caused by different crosslinking conditions, especially changes in function and morphology, in addition to studying the protective effect of ripasudil on endothelial injury to the cornea.

In the present study, we demonstrate that riboflavin, as a photosensitizer, damaged the corneal endothelium's integrity and function under UVA irradiation. The barrier integrity and pump efficiency of the corneal endothelium after combined riboflavin/UVA treatment were destroyed in comparison with the same dose of UVA treatment alone. The corneal endothelium's barrier and pump activities are critical for maintaining corneal transparency [34, 35]. Our results revealed a correlation between variations in these characteristics and CXL-induced edema. In the CXL-0.54 J/cm^2^ group, the corneal endothelium's tight junction and pump activities were recovered to almost the same extent as those in the non-CXL groups, and the transparency of the cornea was also restored by day 14. This reversal indicates that the corneal endothelial function underwent time-dependent recovery. In contrast, the recovery of the CXL-1.08 J/cm^2^ group was incomplete after 14 days. In a previous study, the cytotoxic radiation level following combined riboflavin/UVA treatment was approximately 10 times lower than that after UVA treatment alone since riboflavin enhances the cytotoxic effect caused by the oxidation of UVA light owing to the increase in UVA absorption [4]. After riboflavin treatment, the absorption of UVA in the cornea increased to 95%, while that without riboflavin reached only 25–35% [36]. Pitts et al. [37] discovered corneal endothelial cell injury in pigmented rabbits following a relatively high surface UVA dose of 42.5 J/cm^2^ in the absence of a photosensitizing agent. Our relatively low cytotoxic UVA surface dose of 0.54 J/cm^2^ can be paraphrased by the multiplying effect on UVA absorption by riboflavin [38]. However, UVB-induced endothelial cell loss can be found at a lower dosage level of 0.47 J/cm^2^ in rabbit corneas due to the shorter wavelength of UVB and accordingly higher energy content [39].

We found that *in vivo* treatment with riboflavin and UVA caused oxidative DNA damage in endothelial cells. Riboflavin induced increased expression of *γ*-H2AX and 8-OHdG under UVA irradiation, the extent of which was dependent on the dose of UVA, but this upregulated expression did not occur following treatment with either riboflavin or UVA alone. Reactive oxygen species (ROS), for example, singlet oxygen produced by CXL, are biologically toxic [40]. Excessive ROS levels cause rapid oxidative damage to proteins, cell membranes, mitochondria, and/or nuclear DNA [41]; thus, this can explain why we found corneal endothelial cell DNA damage following combined treatment with riboflavin and UVA. In contrast, UVB is directly absorbed by DNA, especially aromatic heterocyclic bases, which absorb chromophores efficiently, with maximal absorption occurring between 260 and 280 nm [42]. The endothelial layer is more susceptible to the redox imbalance caused by UVA; therefore, changes in the macromolecules of endothelial cells during corneal crosslinking require further study. For example, oxidative damage to cellular components and modification of redox-active proteins are essential for understanding the molecular basis of the oxidative reaction of endothelial damage induced by corneal crosslinking. In comparison with the same dose of UVA or riboflavin treatment alone, UVA combined with riboflavin caused corneal endothelial cell DNA damage, disrupted the tight junction and pump functions of the endothelium, and further induced corneal edema and increased corneal thickness. This suggests that riboflavin is phototoxic to the endothelium during CXL treatment of thin corneas and loses its protective effect at the endothelial level; thus, new crosslinking agents with lower corneal toxicity are required.

We observed that the ROCK inhibitor, ripasudil, exhibited protective effects against damage caused by CXL in corneal endothelial cells. In previous studies, fasudil, which has a structure similar to that of ripasudil, had an indirect antioxidant effect in various disease models such as hypercholesterolemia, diabetes, and ischemia [43–45]. ROS can be generated during corneal crosslinking, which can cause corneal cell damage [46]. Researchers have reported that ripasudil can inhibit oxidative stress and the generation of ROS via the Rho/ROCK pathway in the neuroprotective treatment of glaucoma [47]; therefore, ripasudil may have similar antioxidant effects and thus protect endothelial cells in our CXL model. Our study demonstrates that ROCK inhibition by ripasudil can reduce DNA damage, decrease the destruction of connections between endothelial cells, increase endothelial cell density, and protect the pump and barrier functions of the corneal endothelium. In a previous study, exposure to UVB greatly increased the level of DNA damage, and the blockade of RhoA/ROCK with CT04 or Y27632 could completely inhibit UVB radiation-induced damage [21]. Here, we show that ripasudil can decrease the expression of *γ*-H2AX, which is regarded as a marker of DNA damage. Moreover, we found that ripasudil protected the key functional proteins involved in endothelial pump and barrier functions in the CXL model. Based on the present results, the ROCK inhibitor, ripasudil, decreases endothelial damage induced by corneal crosslinking, indicating mediation via the ROCK signaling pathway; however, the specific mechanism has yet to be elucidated.

There are limitations to this study. Although we found that the protective effect of ROCK inhibitor ripasudil on corneal crosslinking-induced endothelial injury was mediated by ROCK signaling pathway, the specific mechanism was not explained. This is the next step in the future. Secondly, the animal model used in this study is mice, so rabbits can be used for characterization and further study of mechanism to better simulate. There is also more evidence for clinical trials of drugs that are later extended to humans.

## 5. Conclusions

Riboflavin combined with UVA may cause oxidative damage to the corneal endothelium, and the repair of endothelial damage caused by corneal crosslinking is dose-dependent. The ROCK inhibitor, ripasudil, had a protective effect on endothelial cells during corneal crosslinking. These results provide a basis for further investigation into the specific mechanism and treatment of endothelial damage caused by crosslinking.

## Data Availability

All data used to support the findings of this study are available from the corresponding author upon request.
